# Drug screening targeting TREM2-TYROBP transmembrane binding

**DOI:** 10.1186/s10020-025-01229-y

**Published:** 2025-05-05

**Authors:** M. Cobas-Carreño, A. Esteban-Martos, L. Tomas-Gallardo, I. Iribarren, L. Gonzalez-Palma, A. Rivera-Ramos, J. Elena-Guerra, E. Alarcon-Martin, R. Ruiz, M. J. Bravo, J. L. Venero, X. Morató, A. Ruiz, J. L. Royo

**Affiliations:** 1 Departamento de Especialidades Quirúrgicas, Bioquímica e Inmunología, Facultad de Medicina, Universidad de Málaga, Málaga, Spain; 2 Proteomics and Biochemistry Unit, Andalusian Centre for Developmental Biology, CSIC- Pablo de Olavide, University, Seville, Spain; 3 Trinity Biomedical Sciences Institute, School of Chemistry, The University of Dublin, Trinity College, Dublin, Ireland; 4 Instituto de Biomedicina de Sevilla, IBiS/Hospital Universitario Virgen del Rocío, CSIC/Universidad de Sevilla, Sevilla, Spain; 5 Departamento Bioquímica y Biología Molecular, Facultad de Farmacia, Universidad de Sevilla, Seville, Spain; 6 Biggs Institute for Alzheimer’s and Neurodegenerative Diseases, University of Texas Health Science Center, San Antonio, TX USA; 7 Ace Alzheimer Center Barcelona-Universitat Internacional de Catalunya, Barcelona, Spain; 8 Networking Research Center on Neurodegenerative Diseases (CIBERNED), Instituto de Salud Carlos III, Madrid, Spain

**Keywords:** TREM2, DAP12, TYROBP, Drug screening, B2H, BATCH, Varenicline

## Abstract

*TREM2* encodes a microglial membrane receptor involved in the disease-associated microglia (DAM) phenotype whose activation requires the transmembrane interaction with TYROBP. Mutations in *TREM2* represent a high-impact risk factor for Alzheimer’s disease (AD) which turned TREM2 into a significant drug target. We present a bacterial two-hybrid (B2H) system designed for high-throughput screening of modulators for the TREM2-TYROBP transmembrane interaction. In a pilot study, 315 FDA-approved drugs were analyzed to identify potential binding modifiers. Our pipeline includes multiple filtering steps to ensure candidate specificity. The screening suggested two potential candidates that were finally assayed in the human microglial cell line HMC3. Upon stimulation with anti-TREM2 mAb, pSYK/SYK ratios were calculated in the presence of the candidates. As a result, we found that varenicline, a smoking cessation medication, can be considered as a transmembrane agonist of the TREM2-TYROBP interaction.

**Supplementary Information:**

The online version contains supplementary material available at 10.1186/s10020-025-01229-y.

## Introduction

TREM2 (triggering receptor expressed on myeloid cells 2) is a transmembrane receptor expressed in most innate immune cells including microglia and astrocytes. The expression of TREM2 in these cells underscores its key role in maintaining homeostasis in the brain and other tissues, and in regulating inflammatory and repair processes. Glial TREM2-dependent pathway favors the structural integrity and functionality of brain synapses, protecting from the cognitive decline associated to Alzheimer’s disease (AD) (Leng and Edison 2021; Fracassi et al. 2023). Due to the apparent lack of function of its cytoplasmic effect, TREM2 interacts with TYROBP (TYRO protein tyrosine kinase-binding protein), an adaptor protein. The binding of TREM2 to its ligand provokes TYROBP dimerization and the tyrosine phosphorylation of its ITAM (immunoreceptor tyrosine-based activation motif) domains. Recently, it has been proposed that this activation is mediated by SYK (spleen tyrosine kinase)-dependent and -independent pathways (Wang et al. 2022a, b). When ITAM domains are phosphorylated, they recruit SYK, which activates numerous signaling proteins. Among them, we found the phosphadytlinositol 3-kinase (PI3K)-Akt pathway, that ends up activating mTOR, a protein involved in energetic metabolism and protein synthesis. SYK also inactivates GSK3β (glycogen synthase kinase-3β) which promotes cell proliferation. This pathways is responsible of key microglia functions such as phagocytosis, cytokine secretion and lipid metabolism (Jay et al. 2017; Colonna 2023). TREM2 is involved in a wide range of microglial functions ranging from motility and proliferation to energetic metabolism maintenance (Ayyubova 2023; McKee et al. 2023). Different studies suggested that TREM2 could exert an anti-inflammatory action and increased activation of the TREM2 pathway could be associated to a slower cognitive decline (Fracassi et al. 2023). Under this scenario, AL002c was launched, consisting in a mouse IgG1 anti-hTREM2 monoclonal antibody (mAb) generated using the recombinant hTREM2 extracellular domain as an immunogen in a hybridoma approach, followed by humanization and affinity maturation by yeast display (Wang et al. 2020). Shortly after, a humanized version of the monoclonal antibody, was released which counteracts TREM2 decreased functionality by optimizing its signaling to improve cell survival and proliferation, and activity of microglia (Clinical trials.gov/ INVOKE-2). In parallel, other strategies focus on the use of small molecules rather than mAb. It has been recently initiated the Phase I clinical trial of VG-3927, a novel small molecule TREM2 agonist, to treat common neurodegenerative diseases associated with microglia. However, evidence suggests that activated microglia could contribute to neuronal death in advanced stages of the disease suggesting that the TREM2-dependent pathway might act as a double-edged sword in the AD (Konishi H, et al. 2018; Long et al. 2019; Qin et al. 2021; Garcia-Alberca JM et al. 2024). With the objective of increasing our pharmacological arsenal against this therapeutic target, we have developed a high throughput screening strategy focused on the identification of potential modulators of the TREM2-TYROBP interaction. More specifically, we have focused our target to their transmembrane binding, in an attempt to find molecules with high hydrophobicity scores that eventually would facilitate the blood-brain barrier penetrance. Here we describe the use of a bacterial two-hybrid (B2H) system that takes advantage of the transmembrane binding between TREM2 and TYROBP. A bacteria strain is engineered to report the strength of this affinity what allows high throughput selection of both agonists and antagonists of this interaction. We report the results of the pilot screening and validate our candidate with both in vitro and in silico independent approaches. First, checking the biochemical effect in a microglial cell culture and second, studying the structural resolution of the complex with molecular docking analysis.

## Materials and methods

### Plasmids and strains

Constructs contained in the N-terminal site the *Pseudomonas aeruginosa* phage Pf3 coat protein signal peptide (QSVITDVTGQLTAVQADITTIGG) (Kiefer D, et al. 1999) followed by the transmembrane domain (TMD) of the either TYROBP (VLAGIVMG**D**LVLTVLIALAVYFL) or TREM2 (SILLLLACIFLI**K**ILAASALWA), which form the α-helix that constitute the TMD. These are known to contact in their natural context via their aspartic and lysine residues while they align across the glial cellular membrane. Constructs contained a cytoplasmic 3-glycine elbow to increase flexibility in order to facilitate the interaction between the two *Bordetella pertussis* adenylate cyclase complementary domains (T18 and T25) of the BATCH dual hybrid system (Battesti A, et al. 2012). The bacterial two-hybrid system based on adenylate cyclase which generates cAMP what in the *Escherichia coli* BTH101 background turns into increasing beta-galactosidase expression. Plasmids containing the chimeric fusions were ordered from Genscript (https://www.genscript.com/) and further subcloned using conventional molecular biology techniques in DH5α *E. Coli* strain. The complete protein used as bait is translated from pKNT25 of the in-frame cloned BATCH pKTN25 plasmid (Supplementary material) (Table 1).

**Table 1:** Strains and plamids

Strains	
*E. coli* DH5α	fhuA2 lac(del)U169 phoA glnV44 Φ80’ lacZ(del)M15 gyrA96 recA1 relA1 endA1 thi-1 hsdR17.
*E. coli* BTH101	Str^R^. lacZ + strain that responds to intracellular cAMP levels of the BACTH system. Genotype: F-; cya-99, araD139, galE15, galK16, rpsL1 (Str), hsdR2, mcrA1, mcrB1.
Plasmids	
pKNT25-TYROBPTM	Km^R^. Plasmid with the transmembrane domain of human TYROBP fused to the NH_2_- terminus of the T25 fragment of adenylate cyclase together with the signal peptide Pf3. First component of the initial drug screening system.
pUT18-TREM2TM	Ap^R^. Plasmid with the transmembrane domain of human TREM2 fused to the NH_2_- terminus of the T18 fragment of adenylate cyclase together with the signal peptide Pf3. Second component of the initial drug screening system.
pKNT25-zip	Km^R^. Positive control plasmid (ZIP) for the BACTH system. First component of the system for discarding non-specific inhibitors.
pUT18-zip	Ap^R^. Positive control plasmid (ZIP) for the BACTH system. Second component of the system for discarding non-specific inhibitors.
pKNT25	Km^R^. Plasmid backbone prepared to clone at the amino terminus of the T25 fragment of the adenylate cyclase of the BACTH system. First component of the nonspecific activator discard system.
pUT18	Ap. Plasmid backbone prepared to clone at the amino terminus of the T18 fragment of the adenylate cyclase of the BACTH system. Second component of the nonspecific activator discard system.

### Cell culture assays

HMC3 cells (ATCC, Manassas, VA, USA) were maintained in DMEM medium supplemented with 10% fetal bovine serum, GlutaMAX, and 1% penicillin/streptomycin/fungizone (Thermo Fisher Scientific, Waltham, MA, United States). Semiconfluent (50–70%) cultures were stimulated according to prevoulsy described procedure (Nugent AA, et al. 2020). This consisted in an incubation with 1 µg/ml anti-TREM2 (1:1 anti-TREM2 R&D systems AF1729; anti-TREM2 R&D MAB17291) for 5 min at 37ºC in the presence of 1% dimetilsulfoxide (DMSO) alone or in combination with the candidate drug. Plates were then rapidly placed on ice and washed twice with phosphate buffer saline, in situ lysated with TRIsure and stored O/N at -80ºC for at least 24 h. Proteins were fractionated by electrophoresis using 10% sodium dodecyl sulphate (SDS) polyacrylamide gels, electroblotted into PVDF membranes (Hybond-P, GE Healthcare), and blocked with 5% BCA in TBS. Membranes were then incubated with the different antibodies overnight at 4 °C (anti-hSYK #2710, anti-hPhospho-SYK #2712 from Cell Signaling were used according to manufacturer’s instructions), followed by incubation with a horseradish peroxidase-conjugated antibody. The immunoreactive bands were visualized using ECL (Invitrogen). The images were obtained and analyzed with ChemiDoc™ Touch Imaging System (Bio-Rad). Cell-based ELISA for pSYK/SYK ratios were determined using determined with Boster Biological technology (Pleasanton, CA, USA) kit #EKC2006 according to manufacturer’s instructions. Briefly, 20,000 HMC3 cells were plated on a 96-well format for 24 h, and challenged with different concentrations of anti-hTREM2 AL002 (Antibody system #DHJ69201) for 5 min. Cells were then washed and fixed before adding either anti-SYK or anti-TYR525pSYK). Secondary antibodies conjugated with hoseradish peroxidase where used to catalyze the colorimetric reaction as stated in manufacturer’s protocol.

### Semiquantitative bacterial two hybrid

Prior to each assay, plasmids were freshly transformed in *E. coli* BTH101 and plated at 37 ° C overnight (O/N). Four colonies were selected from each plate, added separately to 1 mL of LB with 100 ng/µL ampicillin and 50 ng/µL of kanamycin, and stirred at 1200 rpm overnight at 37° C. The next day, 10 µL drops of each biological replica were seeded in triplicate on plates with both antibiotics and grown at 30 °C. After 24 h, a picture was taken to analyze the intensity of blue of each drop with the ImageJ software (W. Rasband, National Institutes of Health). The blue color correlates with the amount of cAMP generated, linked to the interaction capacity of the study proteins, and was normalized with respect to the negative control (BTH101 with pUT18 and pKNT25). For the analysis, a 32-bit image type was selected, the different rows were defined with the Analyze Gel command, then the baseline was defined with Plot Lanes and the interior of the peak area was chosen to obtain the result.

### Quantitative bacterial two hybrid

Freshly transformed *E. coli* BTH101 with the necessary plasmids were grown O/N at 37 °C in 0.5 mL of LB with both ampicillin 100 µg/ml and kanamycin 20 µg/ml. 1:50 dilutions were grown in 10 mL tubes at 30 °C and 180 rpm until OD_600_ reached 0.2–0.3. Culture was then divided in 200 µl aliquots in flat-bottom 96-well plates where drugs (FDA-Approved Drug Library HY-L022, MedChem Express, Monmouth Junction, USA) were assayed in triplicates at 150 µM. Positive controls contained 150 ng/mL IPTG and DMSO 1%. Plates were sealed to avoid evaporation and incubated O/N at 180 rpm and 30ºC. For the enzymatic assay 100 µL of diluted 1:10 aliquots were taken and OD_620_ was determined in an ELISA reader. 50 µL of these diluted samples were added to 705 µL of Z buffer (60 mM Na_2_HPO_4_ 7H_2_O; 40 mM NaH_2_PO4 H_2_O; 10 mM KCl; 1 mM MgSO_4_ 7H_2_O) with 0.3% of freshly added ß-mercaptoethanol, 30 µL of chloroform and 15 µL of 0.1% SDS. Samples were vortexed for 10 s and stabilized for 2 min in a water bath at 30 °C. The reaction started adding 200 µL ONPG (4 mg / mL in Z buffer) and stopped with 0.5 ml Na_2_CO_3_ 1 M before being placed on ice. Samples were then spined and placed in an ELISA 96-well plate for OD_405_ measurement. ELISA reader filters available were 620 nm and 405 nm instead of the 600 and 420 defining the Miller Units. Correlations between the 600 and 420 nm absorbances taken with a conventional spectrophotometer and the 620 and 405 nm with the ELISA plate reader were R^2^ > 0.98, (p-value < 0.001, Spearman’s test) and allowed us to define the Adapted Miller Units (UMAs) as: 1000×[OD_405_ / (t × 0,05 × OD_620_)] (Supplementary Fig. 1).

### Molecular docking

The 3D structure for Varenicline (DB01273) was obtained from the Drugbank Olie database (Knox et al. 2024) and the TREM2 and TYROBP α-helices were extracted from our previous molecular dynamics simulation (Garcia-Aberca JM et al. 2024). The input files for the docking studies for the helices and the ligand were prepared using the AutoDockTools (Morris et al. 2009) software package and the docking simulation was performed using the AutoDock Vina (Eberhadt J et al. 2021; Trott O et al. 2010) software. The box was set to cover all the inter-helices space (size_x, _y and _z = 36, 32, 86 and center_x, _y, _z = 49.696, 46.055, 32.111) and the energy_range and exhaustiveness parameters were set to 4 kcal/mol and 8 respectively. The results of the docking analysis were visualised using PyMOL software (The PyMOL Molecular Graphics System, Version 1.2r3pre, Schrödinger, LLC, Germany). The NCI establish between the ligand and the two helices were analysed using the NCIPLOT4 software package and visualised using Jmol with a density isososurface of 0.4 and a colour range of [-2,2] with blue representing attractive interactions, red representing repulsive interactions and green representing the weaker interactions.

## Results

**Fig. 1:**
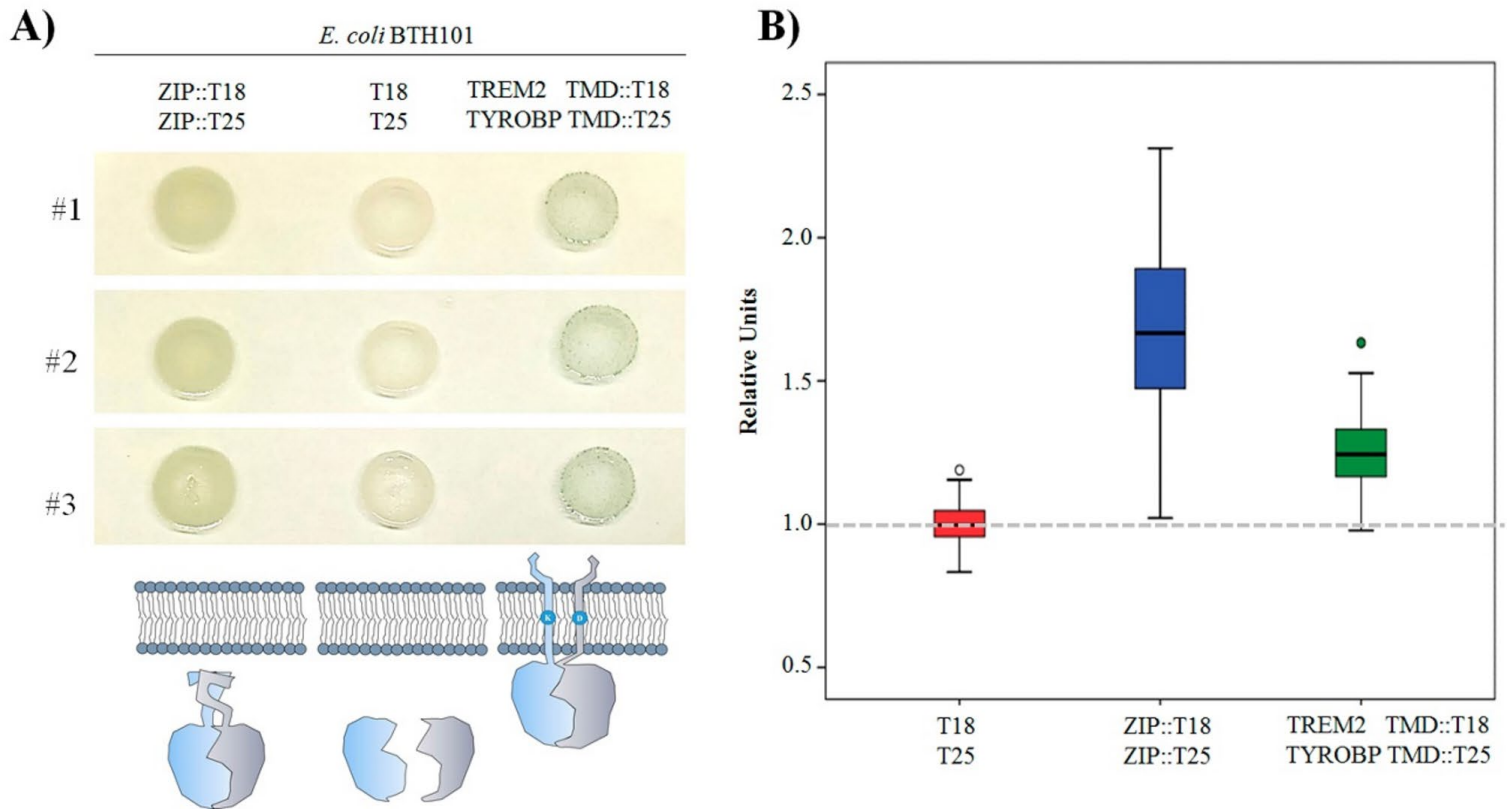
Transmembrane BATCH two hybrid system (**A**) Three independent drops *of E. coli* BTH101 strain harboring different plasmid configurations grown overnight at 30ºC in LB X-gal plates with Km and Ap. ZIP domains allowed a cytoplasmic reconstitution of the adenylate cyclase as the TREM2 and TYROBP TMD do in the membrane turning blue the drops. Color background obtained from the T18 and T25 control plasmids comes from fortuitous cytoplasmic binding. (**B**) Box plot showing median, quartiles and range values of the relative intensities compared to the empty plasmids. Semiquantitative analysis of the X-gal drops under the different configurations (T18/T25 *n* = 99; ZIP::T18/ZIP::T25 *n* = 81; TREM2TMD::T18/TYROBP TMD::T25 *n* = 57)

Drop assays of *E. coli* BTH101 strain harboring different plasmid configurations showed that both human TMDs effectively bind in the bacterial membrane (Mean of 1.4 vs. 1.01, p-value < 0.01, Mann Whitney test), as predicted by dynamic models and according to what previously reported (Fig. 1). However, the dynamic range of this beta-galactosidase assay was lower than expected. For this reason we decided to perform the screening in liquid cultures.

**Fig. 2:**
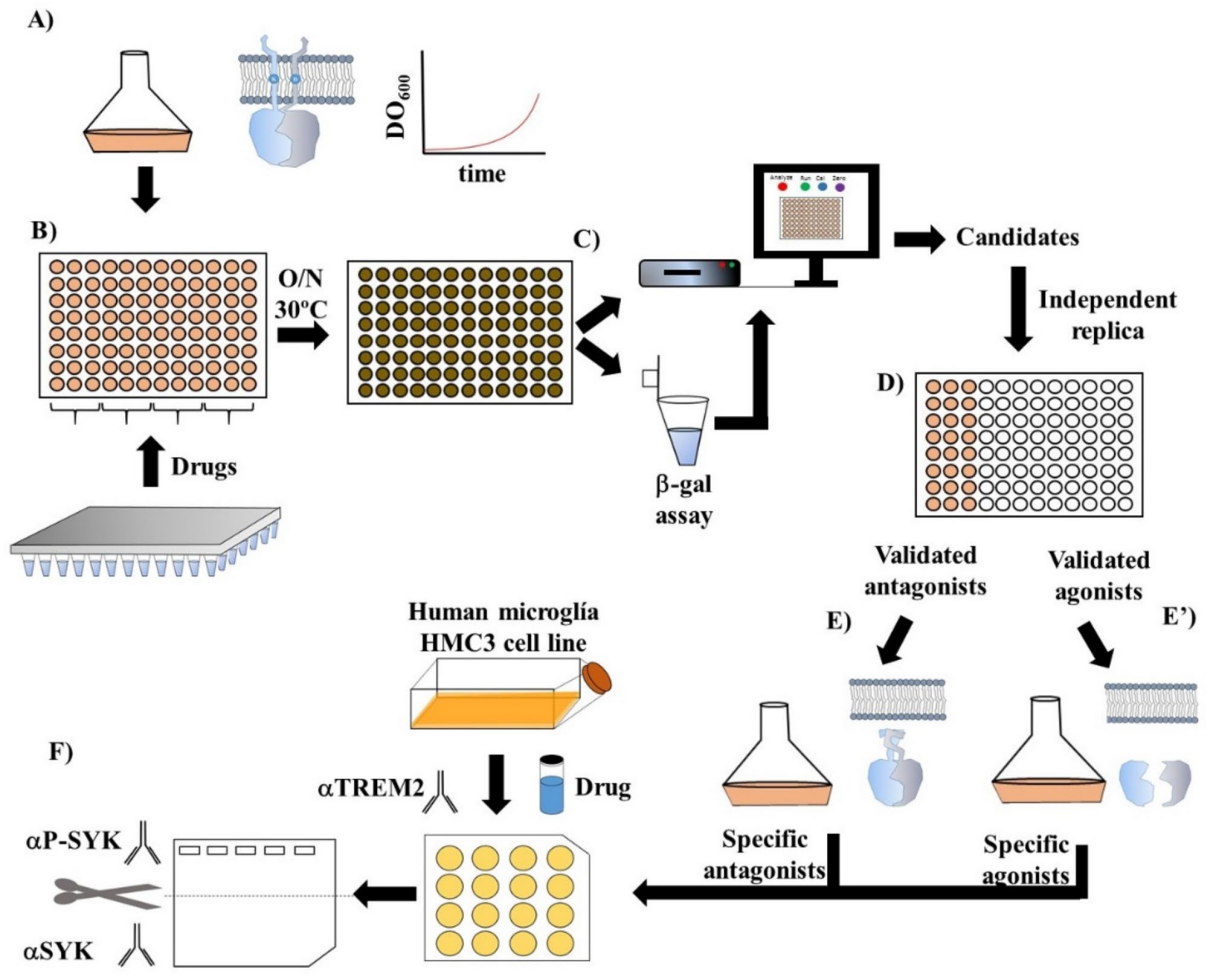
Screening pipeline. **A**) *E. coli* BTH101 bearing the two plasmids with TREM2TMD::T18 and the TYROBPTMD::T25 was cultured until OD_600_ = 0-2-0.25. **B**) Exponential-phase bacteria were distributed into a 96-well plate in combination with the different drugs. Plates were incubated o/n at 30ºC. **C**) Cells were diluted 1:10 and growth was calculated as OD at 620 nm. Beta-galactosidase assay were performed and OD_405_ was also calculated. **D**) Candidates were independently repeated resulting in validated agonists and antagonists. **E**) To determine specificity, true antagonist drugs were challenged to BTH101 stain bearing ZIP::T18 and ZIP::T25. **E’**) similarly, true agonist were challenged to BTH101 strain bearing the empty plasmids producing T18 and T25 plasmids, where any binding should be considered spurious

Thus, we tested 315 FDA-approved drugs which were assayed in triplicate and compared with the negative control (Fig. 2A). We selected the most promising candidates 38 drugs: 25 potential activators and 13 repressors, which represented 8% and 4% of the assayed compounds, respectively. These were individually assayed to perform an independent assay to validat the modulating capacity of 13 of these drugs (10 activators and 3 inhibitors). This bias in the identification of activators (3.2%) vs. inhibitors (1%) was statistically significant (*p* = 0.049, χ^2^ test) (Supplementary Table 1). Statistical analysis showed the results of the 13 validated candidates were significant (p-value = 0.014, Mann Whitney test).

Next, the B2H confirmed candidates underwent a specificity assay. The rationale for the following test is to discard any potential cAMP downstream pathway activation/inhibition beyond the pure TMDs binding. To test the activator specificity, we performed the β-galactosidase assay in the *E. coli* BTH101 strain bearing both plasmids lacking the Pf3::TMD:: 3xGly domains. On the other hand, to test inhibitor specificity, we repeated the enzymatic assay in a BTH101 strain bearing the pUT18-ZIP and pKNT25-ZIP plasmids. These have the cytoplasmic domains in frame fused to the leucine zipper of the GCN4 transcription factor, making the T18-T25 binding highly stable. True activators/inhibitors shall leave the beta-galactosidase activity of these controls unchanged, since any induction or repression in these conditions would mean unspecific stimulation (Fig. 2E and E’).

**Fig. 3:**
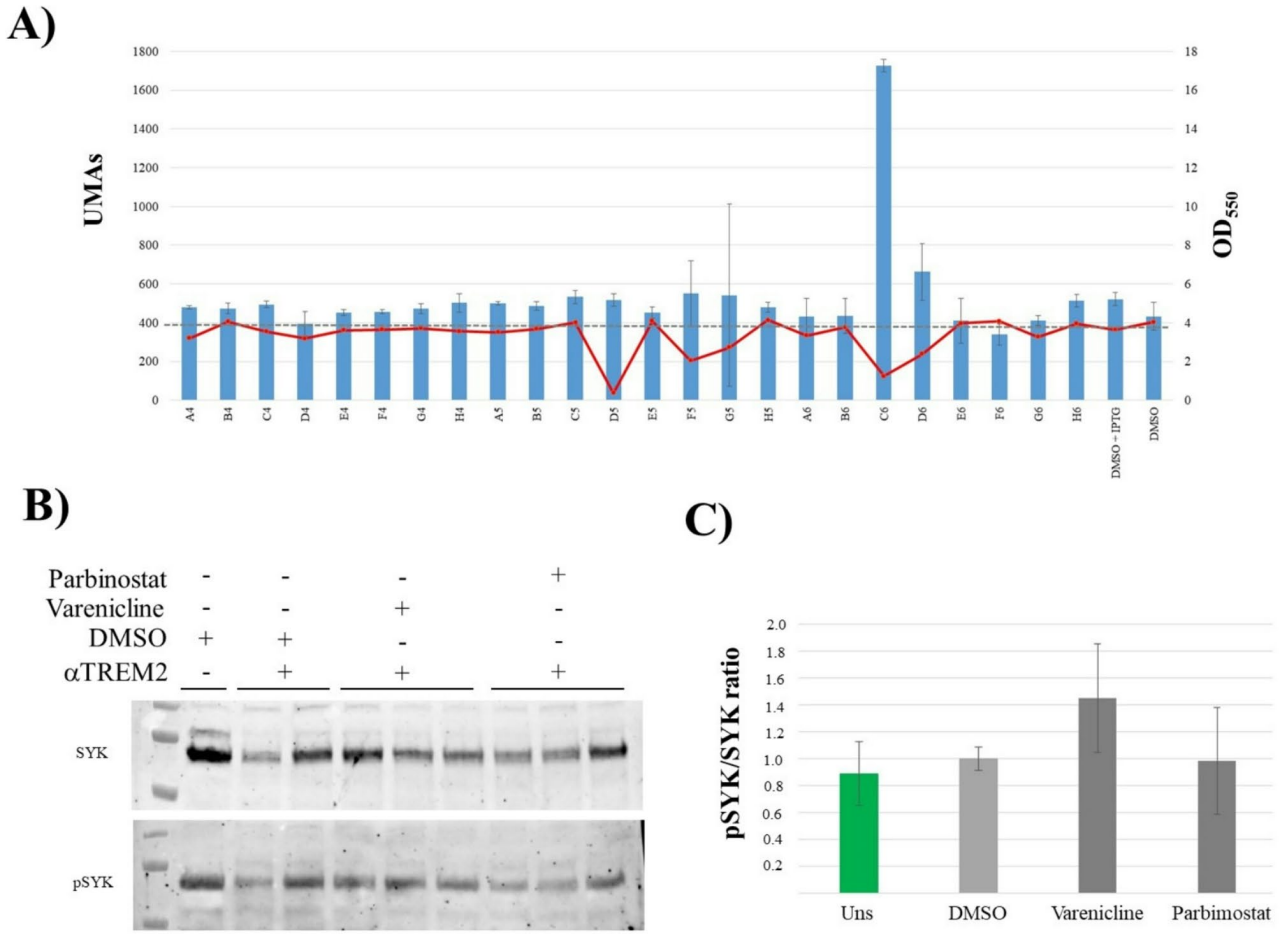
Screening results. (**A**) Barrs showing representative analysis of the beta-galactosidase activity and bacterial growth (red line) of *E. coli* BTH101 with the TREM2TMD::T18 and TYROBPTMD::T25 plasmids after O/N stimulation with the different candidate drugs. Due to the double-blind nature of the assay, compounds were identified by the well position occupied in the drug library. Candidate in well C6 was selected for further characterization. Error bars represent standard deviation (SD). (**B**) Western blot of HMC3 cell culture protein extracts after αTREM2 stimulation with either 1% DMSO, varenicline or parbimostat 150 µM. (**C**) Pathway stimulation after quantifying the different band intensities (*n* = 3)

We found that 3 out of the 13 selected modulators were specific (dexametasone, parbimostat and varenicline), all of them activators. No inhibitors were found to be specific. Next, we selected the two more promising: parbimostat and varenicline, according to their potential for a chronic treatment. Due to its secondary effects, dexamethasone was discarded. These two drugs where assayed in the human cell line HMC3. Cellular TREM2 was stimulated with agonist Ig for a short time in order not to saturate the Syk -to- Phospho-Syk conversion (p-SYK). This challenge was made in the presence of either DMSO, parbimostat or varenicline at 150 µM. No effect was observed with parbimostat which was considered a false positive, our results show that varenicline increased the p-SYK/SYK ratio ≈ 40% (Fig. 3). These results were also independently confirmed using a colorimetric Cell-Based ELISA kit upon the stimulation of different concentrations of AL002 (Supplementary Fig. 3).

**Fig. 4:**
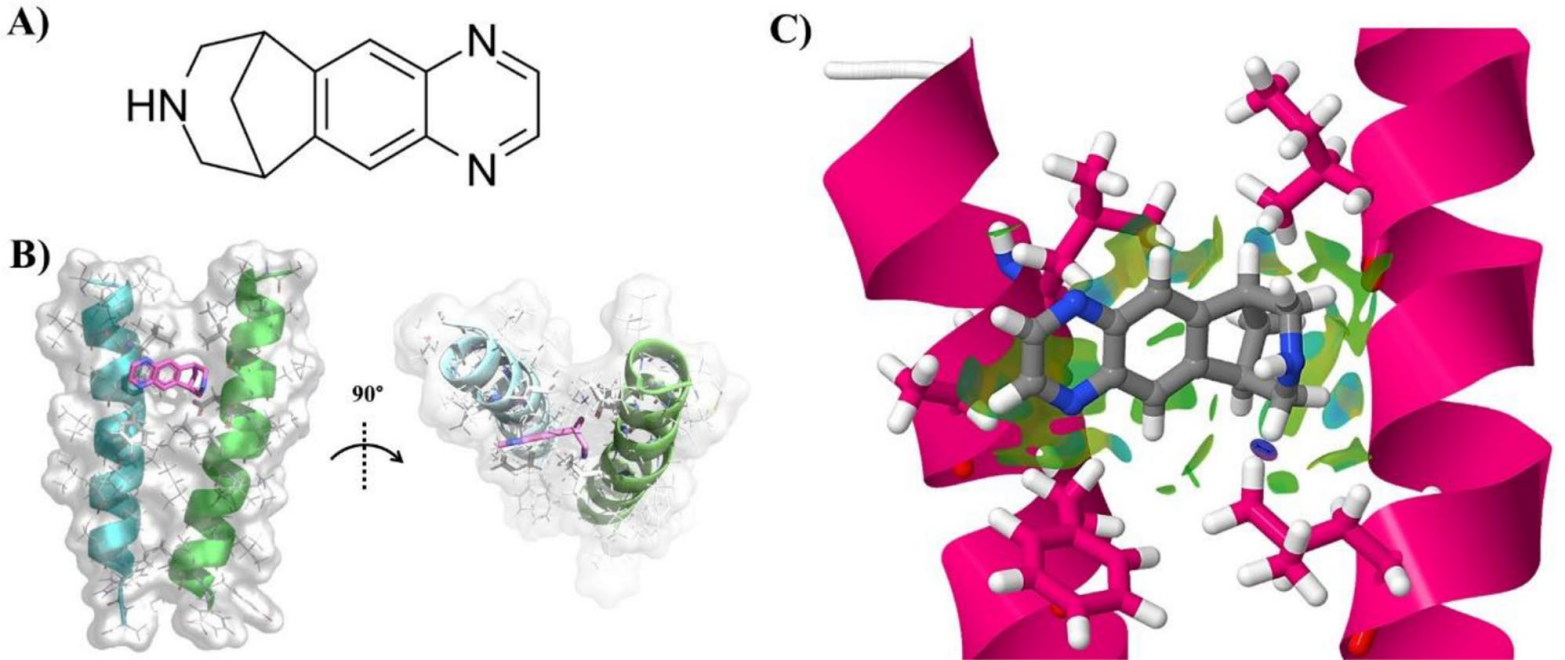
Molecular docking study. (**A**) Varenicline 2D representation. (**B**) Best fitting docking configuration of varenicline with TREM2 (left) and TYROBP (right). (**C**) NCI representation (green area) of the contacts between varenicline and ILE-6 and LEU-13 residues from TYROBP’s TMD (right) and with the LEU-6, ALA-7 and PHE-10 residues in chain TREM2’s TMD (left)

Varenicline is an organic heterotetracyclic compound that acts as a partial agonist for nicotinic cholinergic receptors currently used as an aid to giving up smoking, however its potential role interacting with TREM2 pathway is completely novel. In order to gain further insight into its effect on the TREM2-TYROBP affinity, we tested their interaction in an in silico docking assay. As shown in Fig. 4B, the best scored binding mode presents the varenicline ligand interacting with both α-helices strands with an affinity value of -5.6 kcal/mol. The non-covalent interactions (NCI) analysis shows that the varenicline ligand is establishing attractive interactions with the ILE-6 and LEU-13 residues from TYROBP’s TMD (right) and with the LEU-6, ALA-7 and PHE-10 residues in chain TREM2’s TMD (left) (Fig. 4C).

## Discussion

Variants in the TREM2 gene have been strongly associated with an increased risk of AD, highlighting its crucial role in neuroinflammation and microglial response. TREM2 signaling plays a complex role in Alzheimer’s disease, with its effects varying depending on the disease stage and experimental model used. In APP-transgenic mouse models, impaired TREM2 function leads to reduced microglial activity, which is associated with diminished amyloid-beta clearance. However, in tau-transgenic models, where neuronal damage and death are primarily driven by tau pathology rather than amyloid deposition, knocking out TREM2 or introducing the R47H variant has been shown to mitigate chronic microglial reactivity, which otherwise contributes to neurodegeneration. Thus, the role of TREM2 in AD progression must be carefully contextualized (Long et al. 2019; Qin et al. 2021).

TREM2 has also been shown to interact with other membrane receptors, such as DAP10 (DNAX-activating protein of 10 kDa) (Wang et al. 2022a, b). However, its role in AD is most closely related to its interaction with TYROBP (Zhang et al. 2013). Current drugs such as AL002 target TREM2’s N-terminal region near the extracellular stalk. Here, we postulate that the transmembrane interaction between TREM2 and TYROBP represents a promising pharmacological target for AD, particularly given ability of lipid-soluble small molecules to cross the blood-brain barrier (Pardridge MW, 2012).

The screening of FDA-approved active compounds offers significant advantages. On the one hand, their safety profiles have already been evaluated in humans, though these data must be reassessed in the context of chronic AD treatment as opposed to acute applications. Additionally, manufacturing and regulatory approvals are already in place, facilitating the transition to Phase II clinical trials. Repurposing strategies thus provide a faster, lower-cost, and higher-success-rate approach to drug development.

In this study, we found that varenicline increases TREM2-TYROBP affinity. Varenicline, a partial agonist of the α4β2 subtype of the nicotinic acetylcholine receptor, is currently indicated for smoking cessation. Its potential neuroprotective effects have been suggested in preclinical models of Parkinson’s disease (Bagdas et al. 2018) and in aged non-human primates, where it improved cognition and general performance (Terry et al. 2016). However, previous trials in mild-to-moderate AD failed to demonstrate efficacy (NCT00744978), though this does not rule out potential benefits at earlier disease stages or in larger cohorts (Kim et al. 2014).

Recently, Alector announced results from its INVOKE-2 Phase 2 clinical trial, which evaluated AL002 in early AD patients over 96 weeks. This trial included individuals with confirmed amyloid accumulation, with 67% diagnosed mild cognitive impairment due to AD and 33% with mild dementia (Alector press release, July 2024). These findings emphasize the importance of refining our understanding of microglial function in AD to optimize therapeutic timing and patient selection. If the effect of varenicline on TREM2-TYROBP interaction is further confirmed in vivo, one could speculate the potential benefits of combining both pharmacological strategies targeting the extracellular domain to enhance TREM2 signaling while stabilizing its transmembrane interaction with TYROBP.

The identification of a candidate compound opens the door for rational drug design aimed at optimizing affinity and pharmacokinetics. Our combination of in silico methods and experimental validation, alongside further studies on the molecular interactions described here, will enable the development of compounds with tailored agonist or antagonist effects on the TREM2-TYROBP pathway. We acknowledge certain limitations in our approach. First, hepatic and gastric metabolism may alter the TREM2-TYROBP binding capacity observed in vitro. Second, native TYROBP exists as dimers in microglia, whereas our assays examined monomeric interactions, potentially affecting in vivo relevance. Furthermore, promoting this interaction does not necessarily translate to intracellular upregulation; it may merely extend complex stability or reduce the Km between the two domains. Nonetheless, based on our findings, we are confident that this strategy will allow us to overcome these challenges and achieve promising results.

## Electronic supplementary material

Below is the link to the electronic supplementary material.

Supplementary Material 1

## Data Availability

No datasets were generated or analysed during the current study.
