# Supplementary material for: Drug screening targeting TREM2-TYROBP transmembrane binding
Source: Mol Med. 2025 May 5;31:171. doi: 10.1186/s10020-025-01229-y (PMC12054299; doi:10.1186/s10020-025-01229-y)
Supplement: Supplementary file 1 — Supplementary Material 1 [file 10020_2025_1229_MOESM1_ESM.pdf]

Supplementary files

Drug screening targeting TREM2-TYROBP transmembrane binding

Cobas-Carreño M.[1], Esteban-Martos A.[1], Tomas-Gallardo L.[2], Iribarren I.[3,4], Gonzalez-Palma L.[1], Rivera-Ramos A.[5,6], Elena-Guerra J.[1], Alarcon-Martin E.[1], Ruiz R.[5,6], Bravo M.J.[1], Venero J.L.[5,6], Morató X. [8], Ruiz A.[7,8,9*], Royo J.L.[1,*]

Supplementary results

Supplementary Figure 1. Beta-galactosidase assay absorbance's correlation.

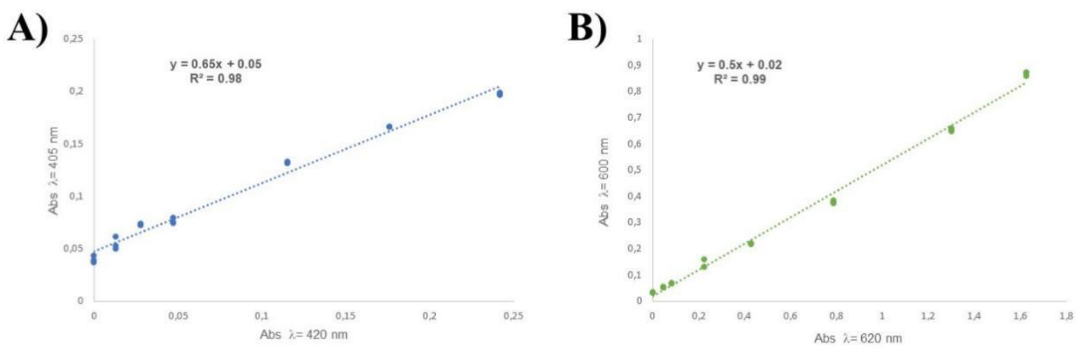

Supplementary Figure 1. Beta-galactosidase assay absorbance's correlation. A) Correlation between absorbance at λ = 420 nm (X axis) and λ = 405 nm (Y axis) taken by the ELISA plate reader. Equation and correlation coefficient (R^2) can be found in the top left corner. B) Correlation between absorbances at λ = 600 nm (X axis) and λ = 620 nm (Y axis) taken by the ELISA plate reader. Equation and correlation coefficient (R^2) can be found in the top left corner. Statistical analysis showed significant results ($p < 0.001$) when performing Spearman's test.

Supplementary Figure 2. Western blot membranes against SYK and pSYK.

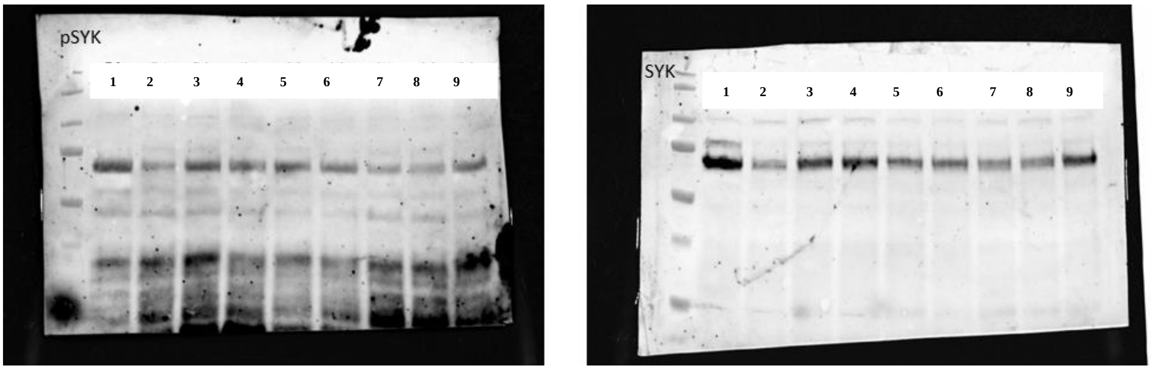

Supplementary Figure 2. Western blot membranes against SYK (A) and pSYK. Proteins extracted from; in lane 1: unstimulated cells with 1% DMSO, lines 2-3: αTREM2 stimulated cells with DMSO, lines 4-6: αTREM2 stimulated cells with varenicline 150 μM and lines 7-9: αTREM2 stimulated cells with parbimostat 150 μM

Supplementary Figure 3. pSYK/SYK ratio measured with cell-based ELISA

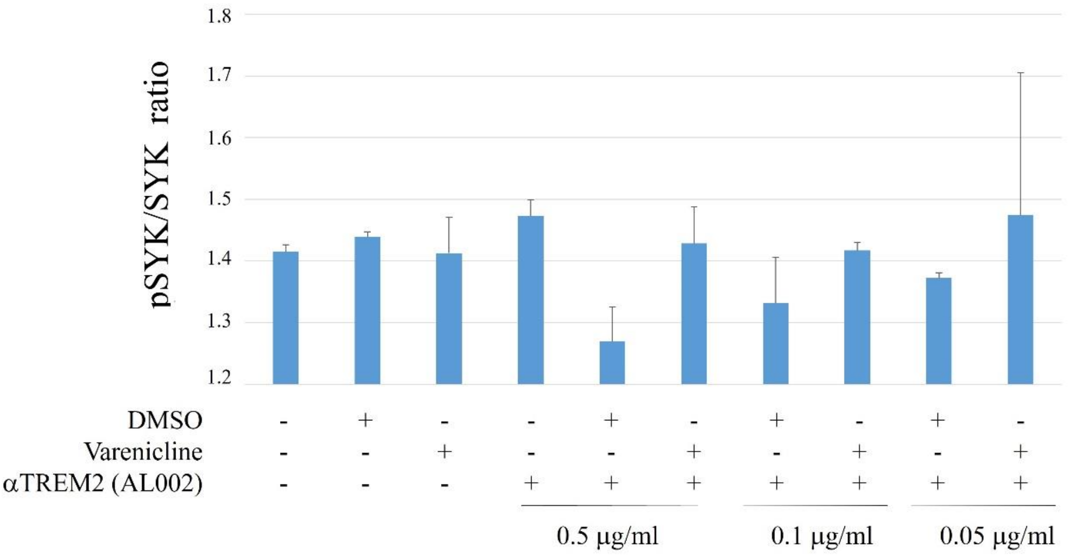

Supplementary Figure 3. pSYK/SYK ratio measured with cell-based ELISA. HCM3 cells were stimulated with different concentrations of AL002 with 1% DMSO or 150 µM Varenicline. Barrs represent averages (n=3) with errors lines the standard deviation.

Antagonists candidates				Agonists candidates			
**Drug**	**Average**	**StdDev**	**B2H confirmation**	**Drug**	**Average**	**StdDev**	**B2H confirmation**
**P163-D9**	0,2	0,03	Yes	**P161-E6**	1,16	0,03	-
**P163-F6**	0,5	0,05	Yes	**P161-H6**	1,17	0,07	-
**P162-G11**	0,5	0,10	-	**P164-G6**	1,17	0,04	-
**P161-A6**	0,68	0,16	-	**P162-E3**	1,18	0,03	-
**P161-G10**	0,74	0,05	Yes	**P162-G6**	1,20	0,03	-
**P162-H8**	0,8	0,18	-	**P162-D4**	1,25	0,07	-
**P162-D5**	0,76	0,05	-	**P163-C6**	1,3	0,25	Yes
**P162-B8**	0,8	0,10	-	**P162-G4**	1,30	0,08	-
**P162-H8**	0,8	0,08	-	**P164-H6**	1,31	0,04	-
**P162-G7**	0,8	0,03	-	**P161-A5**	1,33	0,14	Yes
**P162-B8**	0,8	0,03	-	**P164-H5**	1,36	0,15	-
**P164-E9**	0,90	0,14	-	**P161-E8**	1,36	0,09	-
**P162-D4**	0,9	0,18	-	**P164-G8**	1,45	0,14	-
			-	**P161-F7**	1,46	0,06	-
			-	**P164-H8**	1,49	0,08	-
			-	**P162-F7**	1,5	0,31	Yes
			-	**P164-H4**	1,50	0,14	-
			-	**P164-F10**	1,58	0,04	Yes
			-	**P164-F6**	1,59	0,04	-
			-	**P164-E5**	1,64	0,03	Yes
			-	**P164-C6**	1,66	0,15	Yes
			-	**P161-H4**	1,75	0,25	Yes
			-	**P164-C11**	2,17	0,37	Yes
				**P161-G9**	2,86	0,26	Yes
				**P161-H10**	3,30	1,02	Yes

Supplementary table 1. Initially selected candidate summary. Induction fold -average and standard deviation- of the different candidate drugs. Basal levels were obtained from bacterial cultures in LB with 1% DMSO (average 1.0 + 0.14). As positive control reference, bacterial cultures grown with DMSO plus IPTG was used (average 1.46 + 0.72). Only those independently confirmed in an extra beta-galactosidase assay (B2H confirmation column) underwent the specificity assay.

Supplementary methods

The complete protein used as bait is translated from pKNT25 of the in-frame cloned BATCH pKTN25 plasmid, at the BamHI and HindIII sites, generating the full construct Pf3: TYROBPTMD:

MTMITPSLQSVITDVTGQLTAVQADITTIGGGVLAGIVMGDLVLTVLIALAVYFLGGGDPRVPSS
NSMTMQQSHQAGYANAADRESGIPAAVLDGIKAVAKEKNATLMFRLVNPHSTSLIAEGVATKG
LGVHAKSSDWGLQAGYIPVNPNLSKLFGRAPEVIARADNDVNSSLAHGHTAVDLTLSKERLDYL
RQAGLVTGMADGVVASNHAGYEQFEFRVKETSDGRYAVQYRRKGGDDFEAVKVIGNAAGIPL
TADIDMFAIMPHLSNFRDSARSSVTSGDSVTDYLARTRRAAPSI

The full protein translated as prey from the plasmid pUT18 upon an equivalent cloning generated the Pf3: TREM2TMD construct:

MTMITPSLQSVITDVTGQLTAVQADITTIGGSILLLLACIFLIKILAASALWAGGGDPRVPSSNSAA
SEATGGLDRERIDLLWKIARAGARSAVGTEARRQFRYDGDMNIGVITDFELEVRNALNRRAHAV
GAQDVVQHGTEQNNPFPEADEKIFVVSATGESQMLTRGQLKEYIGQQRGEGYVFYENRAYGVA
GKSLFDDGLGAAPGVPSGRSKFSPDVLETVPASPGLRRPSLGAVERQSI
